# Applications of Cd(Zn)Te Radiation Detectors in Non-Destructive Testing and Evaluation

**DOI:** 10.3390/s25061776

**Published:** 2025-03-13

**Authors:** Anthony R. Whittemore, Elena Maria Zannoni

**Affiliations:** Walker Department of Mechanical Engineering, The University of Texas at Austin, Austin, TX 78712, USA; anthony.w@utexas.edu

**Keywords:** semiconductor radiation detectors, X-ray and gamma-ray detectors, compound semiconductor radiation detectors, high-Z semiconductor radiation detectors, non-destructive testing, NDT

## Abstract

This review explores the applications of room temperature semiconductor detectors, with a focus on Cd(Zn)Te based detection systems, in non-destructive testing and evaluation (NDT&E). Cd(Zn)Te detectors, which operate efficiently at ambient temperatures, eliminate the need for cryogenic cooling systems and offer high energy and spatial resolution, making them ideal for a wide range of NDT&E applications. Key performance parameters such as energy resolution, spatial resolution, time resolution, detector efficiency, and form factor are discussed. The paper highlights the utilization of Cd(Zn)Te detectors in various imaging and spectroscopic applications, including nuclear threat detection and non-proliferation, archaeological NDT, and Unmanned Aerial Vehicle radiological surveying. Cd(Zn)Te detectors hold significant promise in NDT&E due to their high-resolution imaging, superior spectroscopic capabilities, versatility, and portability.

## 1. Introduction

Non-Destructive Testing and Evaluation (NDT&E) is a group of analysis techniques used to evaluate the properties of a material, component or system without causing its damage, alteration or destruction. While NDT focuses on the detection of flaws or defects within a material or structure being examined, NDE provides more in-depth characterization of its condition, determining for example its size, shape, extension and/or the severity of the flaw. These techniques are crucial for the localization and characterization of defects found in complex objects. Radiography and tomographic imaging are the most widely used NDT&E techniques to image defects [1].

**Figure 1:**
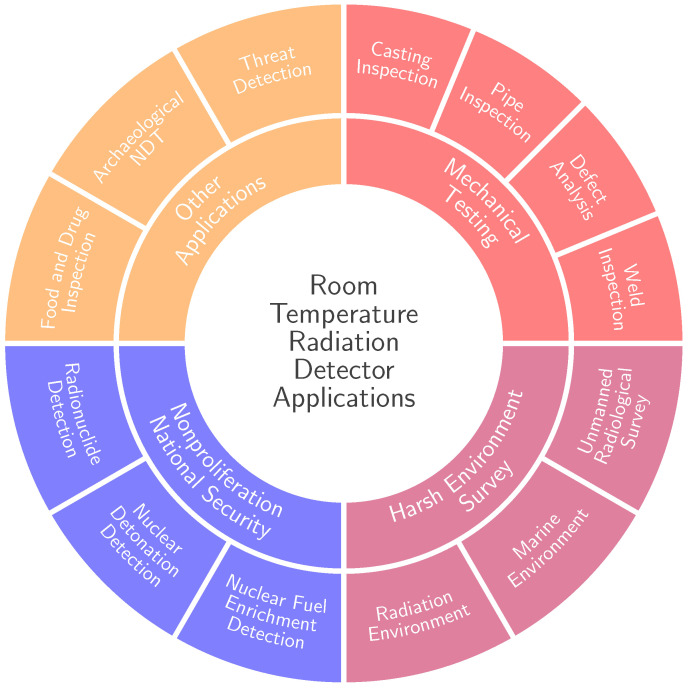
Overview of reported NDT&E applications utilizing RTSDs.

In recent years, room temperature semiconductor detectors (RTSDs) have emerged as a transformative technology finding widespread application across diverse fields, including astrophysics, medical imaging, national security, and NDT&E (Figure 1) [2,3,4,5].

RTSDs, such as Cadmium Telluride (CdTe), Cadmium Zinc Telluride (CZT), Silicon and diamond, operate efficiently at room temperature, eliminating the need for cumbersome and expensive cryogenic cooling systems. This characteristic makes them highly portable and suitable for field applications. Moreover, RTSDs exhibit superior energy resolution and an excellent spatial resolution over their scintillator-based counterparts, enabling precise detection and characterization of defects and material anomalies [6].

While significant advancements have been made in the field of RTSD, a dedicated review focusing on their applications in NDT&E is not currently available. The objective of this paper is to report the recent efforts, key challenges and solutions in using RTSDs for NDT&E, and to highlight their importance in various industries. We will discuss the key specifications of RTSDs and how degrading phenomena affect the overall detector performance. Then, each application (Figure 1) is discussed in detail and categorized as either imaging or spectroscopy focused. This provides both expert and non-expert readers with easy-to-navigate, broad and cross-disciplinary reference materials, and offers insights into emerging trends and future directions.

## 2. Key Parameters of RTSD

**Figure 2:**
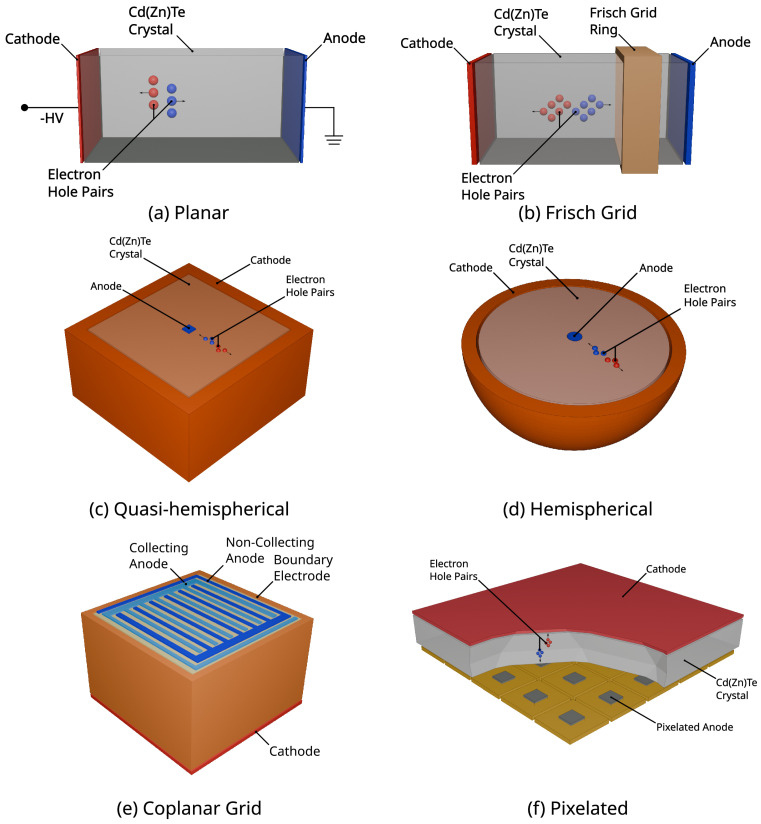
Diagrams of CZT detector configurations reported in literature: (**a**) Planar, (**b**) Frisch grid, (**c**) Quasi-hemispherical, (**d**) Hemispherical, (**e**) Coplanar grid, (**f**) Pixelated. In each configuration the anode and cathode are labeled. Charge carriers are shown: electrons (blue) and holes (red).

Direct and indirect conversion detectors are two primary types of detectors used for high-energy radiation. Direct conversion detectors, such as RTSDs, directly convert the photon hit to an electrical signal. The photon is absorbed into the crystal material through photoelectric effect releasing electrons and creating corresponding electron shell vacancies in the atom, known as electron-hole pairs. The electric field applied across the semiconductor, usually referred to as bias voltage, causes the electrons to drift towards the positive electrode (anode) and the holes towards the negative electrode (cathode) (Figure 2a). The accumulation of charges is then recorded as an electrical signal read by the detector electronics, with the amount of charge collected being proportional to the incoming photon’s energy [7,8]. Additional discussions on detailed semiconductor detector physics can be found in [5,9].

Indirect conversion detectors, on the other hand, measure a photon hit in a two-step process. First, the incident photon interacts with a scintillator material, which converts the high-energy photon into a flash of visible light photons. Second, these light photons are detected by a photosensitive device, such as a photodiode array or a CCD (charge-coupled device), which converts the light into an electrical signal. Phenomena such as photon absorption, light generation, light spread and collection, photoelectron production and collection all introduce uncertainties at each step resulting in a degradation of the detector energy resolution [10].

Direct conversion detectors offer higher spatial resolution and superior spectroscopic performance due to the elimination of subsequent detection processes, while indirect conversion detectors provide higher sensitivity due to thicker absorbing crystals, and faster response times [7,8,9]. The selection between the two types of detectors depends on the specific application requirements and intrinsic detector performance. To facilitate the choice, detector key parameters and inherent trade-offs and degrading factors are summarized in the following sections.

### 2.1. Energy Resolution

The energy resolution is defined as the detector ability to distinguish between different energy levels of incoming radiation. The Full Width at Half Maximum (FWHM) of the energy peak is the primary mode of quantifying the detector energy resolution [4]. There are several factors that contribute in the degradation of the energy resolution in an RTSD, especially in pixelated detectors, namely: Fano statistical noise, electronic noise in the form of detector leakage current, noise from the front-end electronics, and from coupling capacitance, as well as charge sharing, charge loss and the ballistic deficit [11,12]. Visually, the degradation of energy resolution is observed through wider photopeaks and lower energy tails in the signal distribution [13].

The Fano statistical noise represents a fundamental limit to the energy resolution in an RTSD, and it arises from the statistical fluctuations in the number of electron-hole pairs generated by an incident photon [14]. The Fano noise sets a theoretical limit on how precisely the energy of an incoming photon can be determined, even with perfect charge collection and minimal electronic noise. In Table 1, we report the energy resolution of state-of-the-art CZT detectors at different energies, along with their associated Fano noise contributions [15,16,17].

#### 2.1.1. Leakage Current

Leakage current is defined as the unwanted current flowing through the detector in the absence of radiation. The leakage current acts as a source of noise, deemed dark current noise, which reduces the detector’s ability to resolve small amounts of radiation [18]. The dark current noise acts as a signal floor that reduces the overall difference between the electrical signal during detection and when no radiation is present [13]. The smaller the noise floor, the greater the capability to detect small amounts of radiation and to precisely determine the true energy of the incoming photon. The amount of leakage current experienced depends on several factors such as crystal quality, the type of electrode contacts used, and the specific configuration of the detector. High quality CZT detectors typically have a leakage current per pixel ranging from a few to tens of nanoamperes (nA). For example, a 0.5 cm thick CZT detector with a detector area of 2 × $2\;{\mathrm{cm}}^{2}$ containing 8 × 8 anode pixels has a leakage current <0.2 nA/pixel at a bias voltage up to −1500 V [19].

#### 2.1.2. Charge Sharing in Energy Resolution Degradation

During photon absorption, a charge cloud is induced inside the sensor material. The cloud expands through diffusion proportional to the applied bias voltage and the crystal thickness. When the spatial extent of the charge cloud encompasses and is detected by more than one pixel, this effect is known as charge sharing [5,20,21]. This degrades energy resolution since the photon’s energy is distributed among multiple pixels, leading to inaccuracies in the single-pixel spectrum and overall broadened energy peaks. The phenomenon is translatable also to the strip detector configuration, as studied in [22,23].

Common charge-sharing correction methods are charge-sharing addition (CSA) and charge-sharing discrimination (CSD) [20]. In CSA, the energy deposited in each pixel involved in the shared event is summed together and assigned to the pixel with the maximum energy deposition. Contrary to this, CSD simply removes any shared events, recovering the intrinsic spectroscopic performance but strongly reducing the detection capabilities of the detector.

#### 2.1.3. Charge Loss

Charge loss is an umbrella term that encompasses multiple forms of loss of signal. Charge trapping, charge recombination, and inter-pixel charge loss are examples of charge loss in RTSDs. Charge trapping occurs when semiconductor crystals suffer from imperfections introduced during manufacturing or through radiation damage. The defects in the crystal, such as impurity atoms, vacancies, or structural irregularities, introduce localized energy states within the semiconductor material. These energy levels can trap the moving charge carriers as they travel towards the electrodes, preventing them from being collected and contributing to the signal [13].

Charge recombination refers to the process where the induced electron-hole pairs generated by incoming radiation recombine with each other, effectively neutralizing the charge and reducing the signal produced by the detector. Impurity levels in the crystal, strength and uniformity of the applied electric field, and charge carrier lifetime and mobility are factors that affect charge recombination [24].

Inter-pixel charge loss is a mode of incomplete charge collection that occurs in tandem with charge sharing. A portion of the total charge is lost because of a physical gap between pixels, known as inter-pixel gap, that does not collect the induced charges [25,26].

### 2.2. Spatial Resolution

The spatial resolution is defined as the ability to differentiate between two closely spaced features within the detector’s field of view (FOV). Spatial resolution can further be characterized by either the intrinsic spatial resolution of the detector, or the extrinsic spatial resolution of the detection system. The intrinsic spatial resolution is the detector ability to localize individual photon interactions, and it usually matches with the detector pixel size [27] even if sub-pixel resolution approaches have been proposed [28]. On the other side, the extrinsic spatial resolution quantifies the spatial resolution of the complete detection system, including factors such as scatter, collimation, and detector response [29], as detailed in the following subsections. Typically, the spatial resolution is expressed in terms of length such as µm or mm.

A special mention is required for the Depth of Interaction (DOI) information in 3D-sensitive RTSDs. DOI refers to the specific depth within the detector crystal where the incoming radiation interacts and generates electron-hole pairs [30,31]. Correction methods based on the DOI of the photon have been implemented to correct for charge trapping at various depths within the crystal due to material non-uniformity and nonhomogeneous electric field [32]. More robust charge sharing correction methods have also been proposed using accurate drift models that incorporate the DOI as a measure to improve spatial resolution [33,34,35,36].

#### 2.2.1. Charge Sharing in Spatial Resolution Degradation

The spatial resolution of a RTSD detector improves with decreasing detector pixel size. However, when the pixel size becomes considerably smaller than the charge cloud diameter, charge sharing effects degrade the overall detector spatial resolution, blurring what would be sharp edges in an image. The larger the induced charge cloud from higher photon energies, and/or the larger the distance the charge travels in thicker crystals, the more smearing occurs [37]. Several approaches have been proposed to mitigate this effect, including weighted mean pixel clustering to achieve sub-pixel resolution [38,39].

#### 2.2.2. Scatter

Higher energy photons have an increased likelihood of inducing secondary scatter events within the detector material. These secondary Compton scatter interactions cause photon interactions to be recorded at locations different from the initial interaction sites. This phenomenon manifests as a blurring of the sharply defined edges of objects in the resultant image. To mitigate this blurring effect, scatter rejection techniques can be employed by setting a specific energy threshold on the RTSD, thereby minimizing the detection of low-energy scattered photons. The superior energy resolution of RTSDs enhances their ability to effectively reject low-energy scattered photons [40].

### 2.3. Time Resolution

Two key timing parameters for radiation detectors are time resolution and dead time. Time resolution refers to the ability to precisely measure the moment a single radiation interaction occurs based on the generated electronic signal [41,42]. Dead time is defined as the minimum time interval required for the detector to differentiate between two closely spaced events in time [43]. Time resolution depends on factors such as the height and slope of the signal’s leading edge, noise, and the electrode configuration’s weighting potential. Dead time, on the other hand, is influenced by signal height and pulse-shaping time. Both quantities are typically measured in units of time, like nanoseconds (ns) or microseconds (µs). In some cases, time resolution is also expressed in terms of frequency, such as counts per second (cps) in counting applications or frames per second (fps) when describing image acquisition speed. Fast time resolution and low dead time are crucial for high-flux applications where precise timing of radiation events is essential, such as in particle physics experiments or medical imaging applications.

### 2.4. Detector Efficiency

Detector efficiency refers to the probability that a high-energy particle or photon interacts with the detector producing a detectable signal. The geometric and material considerations must be included when discussing the overall detector’s efficiency [5].

The material, or intrinsic, efficiency is determined by the properties of the crystal material. High atomic number materials lead to higher efficiency since such nuclei present larger targets for photon interactions [5]. This clarifies why compound materials such as CdTe and CZT ($Z_{Cd}$ = 48, $Z_{Zn}$ = 30, $Z_{Te}$ = 52) are preferred over Si (Z = 14) and Ge (Z = 32) for detection of higher energy photons.

The geometric, or extrinsic, efficiency depends on the detector’s physical dimensions, including thickness, and geometry. It represents the fraction of emitted radiation that the detector intercepts. This is quantified by the detector’s solid angle, which describes the detector’s apparent size from the perspective of the radiation source [44].

### 2.5. Form Factor

The form factor refers to the detector’s physical dimensions, such as size, thickness, and overall geometry. While RTSDs offer small and compact form factors that allow them to be portable and used in various operating environments, this benefit often necessitates a trade-off in terms of detector geometric efficiency. Achieving high resolution while providing large-scale assemblies indeed remains a significant challenge for RTSDs. This is primarily constrained by the current limitations of crystal growth techniques, which often fail to produce large and high-quality crystals with consistent performance characteristics. A smaller manufactured size alleviates the percentage of crystal non-uniformities that would lead to charge trapping [3,4]. The current solution is to use a modularity approach where smaller detector units are tiled together in larger panels to increase the effective detection area [45,46]. Specifically, each detection unit should offer all 4, or at least 3, sides for effective tiling [46]. Crystal grading schemes and calibration methods are used to assure that the tiled detector units cohesively operate at peak performance [47].

### 2.6. Electrode Designs to Alleviate Degradation Factors

Researchers have explored and proposed various detector designs to mitigate degradation factors in RTSDs [4]. Figure 2 summarizes the most common electrode configurations, namely planar, Frisch grid, coplanar, hemispherical, quasi-hemispherical, and pixelated.

In a planar configuration (Figure 2a), the crystal is sandwiched between two opposite planar contacts serving as electrodes, i.e., anode and cathode. The planar configuration offers a straightforward design, making it easier to fabricate and less expensive compared to more complex configurations. While it assures a relatively uniform electric field across the detector volume, its performance can be limited by charge trapping effects, which can degrade energy resolution due to incomplete charge collection [48].

In the Frisch grid configuration (Figure 2b), a grid or ring is affixed around the crystal near the anode, thereby dividing the crystal volume into an interaction region and a measurement region [49,50]. The interaction region, situated between the cathode and the grid, is where photon interactions and the resulting induced charge cloud occur. Charge carriers then migrate to their respective electrodes and are collected in a manner analogous to the planar configuration. The induced charge at the anode remains zero until the electrons traverse the ring into the measurement region, where the induced charge is subsequently collected. According to the Shockley-Ramo theorem, the induced charge is proportional to the distance traveled by the electron from the grid to the anode [51]. This design mitigates the issue of slow hole mobility, which is problematic in dual charge carrier sensing, but it sacrifices DOI information. Finally, in this configuration there are three voltage potentials applied, one at the cathode, one at the anode, and one at the grid. Each potential must be tuned such that adequate charge mobility occurs without inducing strong surface leakage current which degrades the energy resolution [49,50].

In the coplanar grid design (Figure 2e), two interdigitated anode grids set at different potentials and one continuous cathode are used. The anode grid with the higher potential is known as the collecting anode where the induced charge depends both on electrons and holes, while the lower potential anode is the non-collecting anode and its signal is only affected by the hole transit. The subtraction of the non-collecting anode signal from the collecting anode one generates a signal independent on the hole movement. However, due to electron trapping, the number of electrons collected in the collecting anode is influenced by the DOI, resulting in a depth-dependent subtracted signal. Significant depth-dependent signal variation occurs when there is a large difference in collection efficiencies between electrons and holes (due to mobility differences) and when the hole collection distance is small compared to the detector thickness [52,53,54,55,56].

Hemispherical and quasi-hemispherical designs (Figure 2c,d) are single charge carrier collection methods proposed to optimize the electric field and weighting potential distribution within the detector. These designs create a strong electric field and maximize the weighting potential at the anode, which facilitates efficient collection of the charge carriers produced by radiation interactions [57]. The hemispherical configuration (Figure 2d) typically involves a single anode placed at the center of a hemispherical crystal, with the cathode covering the outer surface. This setup ensures a radial electric field that directs charge carriers towards the anode, enhancing charge collection efficiency and energy resolution [57,58,59,60]. Quasi-hemispherical detectors (Figure 2e), on the other hand, use a similar principle but with a conventional crystal shape having the anode at the center and a segmented cathode on the outer surface. Similarly, this configuration creates a strong electric field near the anode and focuses the weighting potential towards it, thereby reducing the impact of hole transport [57,58,59].

In a pixelated structure configuration (Figure 2f), the anode is divided into an array of small pixels, each functioning as an individual detection unit while the cathode is a continuous planar contact on the opposite side of the crystal. When radiation interacts with the crystal, each pixel collects the charge generated within its vicinity, and the signals from all pixels are processed to reconstruct the radiation event. The design and manufacturing process is more complex and costly, and the segmentation of the anode can introduce additional electronic noise. Additionally, pixelated detectors often require more power for readout electronics, which can be a limitation in portable or battery-operated devices. Despite these challenges, pixelated detectors are highly effective for applications requiring high energy and spatial resolution, making them the preferred choice for imaging applications [61,62,63,64].

## 3. Applications

RTSDs have been widely adopted in the medical industry, specifically in photon-counting Computed Tomography (CT) or in Single Photon Emission Computed Tomography (SPECT) systems [65,66,67,68,69,70,71]. Detector applications in the medical sector require them to be robust in their handling and reliable in their accuracy to provide the most precise diagnostic information for patients. Building on their success in medical applications, these detectors have the potential to make a significant impact in the NDT&E field.

This section explores the diverse NDT&E applications of RTSDs (Figure 1), focusing specifically on Cd(Zn)Te detectors, broadly categorized by their primary function - either to produce high-quality images (imaging) or to perform precise spectroscopic analysis (spectroscopy). In imaging, the output from the inspection is a two or three dimensional visual representation of the object under examination, while the spectroscopic output consists of a one dimensional graphical distribution of the detected photon energies, known as energy spectrum, emitted by a radiation source, transmitted through and/or scattered by the object under investigation.

Applications ranging from nuclear plant pipe inspection to unmanned aerial vehicle nuclear surveying are just some of the interesting applications that have been documented.

### 3.1. Imaging

#### 3.1.1. Real Time Mechanical Tensile Testing

The acquisition speed of Cd(Zn)Te detectors provides the capability of capturing dynamic phenomena during mechanical testing within a single CT scan [72]. While transient effects may occur in detectors at short time intervals and may affect their performance, the high acquisition speeds allow for the possibility of in situ testing. A time-lapse CT scan was performed using a Timepix hybrid detector [73] with an actuation speed of 42 frames-per-second. This equated to a full 400 projection CT scan in 50 s. The detector used was comprised of 5 chip tiles each with 256 × 256 pixels with a 1 mm thick CdTe sensor. The sensitive area was 70.4 mm × 14 mm (1280 × 256 pixels) and the pixel pitch was 55 µm with a minimal detectable energy of 5 keV. The authors recorded the crack formation and propagation in a silicate matrix subjected to three-point bending over 11 total CT scans in under 10 min [72]. The detector temporal effects were corrected frame by frame through a flat field correction method that accounted for crystal deformities, detector charge accumulation effects, and x-ray beam irregularities. The CdTe detector proved that a CT scan with 400 projections under 1 min is possible. The detector also allowed for each projection to have sufficient statistics and contrast with no motion artifacts using the standard filtered-back projection reconstruction method [72].

#### 3.1.2. Carbon Fiber Reinforced Monitoring

RTSDs were compared to film in terms of on-site monitoring capabilities and their ability to detect cracks in carbon fiber rope [74,75]. Digital and film radiography techniques were compared to 3D CT techniques to observe the difference in dynamic range (evaluable minimal to maximal wall thickness) and minimal detectable features in each specimen. The radiographs were both captured on film and using a Modupix RTSD developed by ADVACAM (Prague, Czech Republic) with a 300 µm Si sensor, a pixel size of 55 µm, and a detector area of 25.6 mm × 25.6 mm. The authors investigated Laminography as an alternative tomography technique, where a tilted rotation axis is used to generate 3D images of flat objects [74,76]. Laminography was performed using a Flite 2x1 RTSD developed by X Counter AB (Stockholm, Sweden) using a 700 µm CdTe sensor with a pixel size of 100 µm and a detector area of 102.4 mm × 51.2 mm. Traditional CT was also performed using a Zeiss XRadia Versa 520 system. Although both digital and film radiography techniques were able to determine each of the flaws in the samples under investigation, the 2D radiographs were not able to resolve small delaminations. Both types of radiographs were then compared to the reconstructed images from the Laminography and CT techniques. This allowed the authors not only to view defects and delaminations in the carbon fiber rope but also to determine the volume of these defects. The signal-to-noise ratio (SNR) was much higher using the Modupix detector over film ($SNR_{film}\approx 280$ and $SNR_{DRpcd}\approx 480$) but no depth information can be obtained from the radiographs. The tomography techniques were compared on the basis of scan speed and data fidelity trade-offs. Although the full CT technique provided fiber-level resolution, the total scan time of 11 h did not provide the ability to perform on-site monitoring like the laminography technique. The laminography technique allowed for a 16 × reduction in scan time providing depth information and minimal detectable delamination features of ≥20 µm [74].

Comparing scintillation-based energy integrating detectors to RTSDs, Tartare et al. were able to separate the carbon fiber from the resin in radiographs [75]. The carbon and resin attenuation coefficients were virtually identical at energies above 50 keV, but simulating a semiconductor detector paired with a dual energy two material decomposition method they were able to distinguish between the carbon fiber and resin and provide an approximation of the material thickness [77]. The standard deviations of the thickness measurements for the carbon fiber and resin components in the reconstructed radiographs were 0.03 cm and 0.02 cm respectively [75].

#### 3.1.3. Inspection of Pipes

The portability and image quality of film make it an obvious choice for in field inspections [78]. CdTe detectors can be comparable due to their compact form factor and small pixel size. In [79] a CdTe detector system was used to perform in-service inspection of steel welds. The detector had a sensitive area of 100 × $50\;{\mathrm{mm}}^{2}$ and a pixel size of 100 µm. The authors were able to reduce scatter and noise while increasing the contrast resulting in the minimal detectable defect size of 25 µm at a depth of 100 mm in a double walled steel pipe [79].

The detection of corrosion under insulation in steel pipes were investigated with a radiography based Pipe Corrosion Under Insulation (CUI) profiler [80]. Aluminum and steel pipes with fiberglass/calcium silicate insulation were inspected with a dual-beam gamma-ray absorption technique utilizing two CZT detectors. Insulation thicknesses of 25, 40, and 50 mm were inspected along with pipe internal diameters ranging from 50 mm to 150 mm. Two 100 mCi Am-141 sources were collimated and coupled with two separate CZT detectors with a crystal volume of 20 × 20 × $10\;{\mathrm{mm}}^{3}$ each [80]. The instrument effectively measures changes in the apparent diameter due to the placement of the two detectors on diametrically opposing surfaces of the pipe. The count rates are proportional to the pipe thickness based on the geometry of the non-corroded pipe, the activity of the source, and the material of the attenuating material. The observed changes in count rates would signal an internal diameter change. The device reported diameter deviations every second up to 0.1 mm accuracy, and the lateral scan speed was approximately 6 m per minute [80]. The carriage that housed the source and detectors to the pipe weighed approximately 5 kg and operated on batteries with an approximate battery life of 100 operating hours. The setup was portable both in size and weight and was effective in determining external corrosion under insulation of steel pipes [80].

Similarly, the authors in [81] explored the use of a CdTe-CMOS detector to inspect pipe wall thickness. They tested a mobile pipe inspection system for its radiographic capabilities with a double wall single image technique. The detector used was manufactured by XCounter and had a sensitive area of 50 × $100\;{\mathrm{mm}}^{2}$ with a pixel size of 100 µm [79]. They compared its performance, adhering to ISO 17636-2 image quality standard, to film [82]. They used duplex wire Image Quality Indicators (IQI) stated in the ISO standard and exceeded the requirements. The images achieved an increase in contrast and spatial resolution through 70 mm of steel. They achieved an SNR of 107 which is greater than the required of 70, detected wire diameter of 0.32 mm, and wire separation of 0.1 mm [81].

#### 3.1.4. Automotive Inline Welding Inspection

Schromm et al. [29] investigated all aspects of currently viable non-destructive inspection methods for riveted joints used in the automotive industry. They performed several experiments comparing various x-ray source types, digital detectors, imaging parameters, and scanning strategies. The authors performed a base line scan of a riveted joint with a standard commercial CT setup having a XWT-160-CT X-ray Worx micro-focus tube and an energy integrating flat panel detector, and they compared it with a synchrotron source. The experiments were conducted at the P07 beamline at the Helmholtz-Zentrum Hereon at the Deutsches Elektronen Synchrotron (DESY) synchrotron facility in Hamburg, Germany. The RTSD used in the study was an XC-THOR (CdTe-based) manufactured by Varex Imaging with a pixel size of 100 µm. The scintillator based detector was designed by a collaboration between Helmholtz-Zentrum Hereon and the Karlsruhe Institute of Technology (KIT). It comprised of a $CdWO_{4}$ scintillator that was 100 µm thick coupled to a lens (POG Präzisionsoptik Gera GmbH, Loebichau, Germany) that provided a 10 × magnification and a CMOS sensor (CMV20000 from CMOSIS Imaging Sensors, Antwerpen, Belgium) that featured a 20 megapixel resolution and a pixel pitch of 6 µm. They noted that the beam hardening artifacts that occurred were because of the lack of x-ray penetration with accelerated voltages from 70 to 230 kV. They described that the use of a synchrotron beam source provided superior image quality to that of the commercial CT setup, but it only was feasible with small well cut samples. The 2.5 µm voxel size from the scintillator system paired with the synchrotron x-ray source was not matched but the reduction of beam hardening by energy gating was beneficial. They did note that the CdTe-based detector needed longer exposure time to achieve good image quality. They also acknowledged that by using the CdTe detector they could achieve sufficient image quality using lower accelerating voltages [29].

#### 3.1.5. Casting Inspection

A qualitative and quantitative study was performed to determine the effectiveness of using a RTSD in the inspection of Aluminum cast parts. The qualitative study used a CdTe flat panel detector developed by ANseeN Inc. with a 100 µm pixel size and a sensitive area of 252 × 1024 pixels [83]. The quantitative study used a 64 pixel CdTe linear array detector developed by Hamamatsu with a 1 mm pixel size. Bandara et al. [83] implanted a Super Sealant Polymer Resin (P601) inside of an aluminum casted cuboid sample. They investigated the resolvability of the implanted defect in the part through a quantitative dual-energy CT method. The effective atomic number was modeled as a ratio between the linear attenuation coefficients collected at two distinct energy levels. The estimated effective atomic number calculated through the dual-energy CT model agreed with the P601 sample within 2.92% [83]. They showed that the CdTe detector provided high resolution CT tomographs in less than 10 min. The authors concluded that with advanced CdTe flat panel detectors speedier and more accurate defect detection in the die-casting alloys can be achieved [83].

#### 3.1.6. Threat Detection

##### Explosive Detection

Fey et al. [84] investigated the spectroscopic performance of a RTSD-based gamma camera system by altering the crystal thickness and pixel pitch. The thickness of the CZT crystals used were 0.45, 1, 2, 3 mm each bonded to a Timepix1 Application-specific integrated circuit (ASIC) [73]. The pixel pitches tested were the intrinsic 55 µm and 110 µm obtained by binning 2 × 2 pixels to compare the spectral performance. The metrics of the detector system under investigation were the attenuation efficiency and energy resolution. Higher energy resolution was experienced with increased crystal thickness and larger pixel pitch, shown as an increase in count rate by 1.25 × compared to the 2 mm and 1.84 × compared to the 1 mm thick crystal [84]. This is due to the fact that thicker crystals present a higher probability of absorbing incident gamma rays, which increases the detection efficiency, and larger pixels alleviate charge sharing effects. The larger crystal thickness also allowed the camera system to detect foreign nuclear material at a farther distance away from the source, e.g., 2.8 GBq Co-60 source 20 m away [84].

X-Ray Diffraction (XRD) is a non-destructive analytical technique used to determine the structural information of materials, including their chemical composition, crystal structure, and phase composition [85]. X-rays scatter at specific angles proportional to the crystalline structure of the material. The scatter angles from a single x-ray source is recorded with an array of detector elements. A larger detector array allows wider angles to be captured and more signal to be gathered to generate a more representative spectrum of x-ray energies. Phantom samples are used to to create a database of spectra that then can be compared in real time to determine unknown materials. In 2013, Kosciesza, Schlomka et al. [86] utilized a CZT-based XRD system to determine the presence of explosive materials in luggages, stating that only XRD baggage-scanning systems based on High Purity Germanium (HPGe) detectors have been commercially available. RTSDs like CZT prove to be more advantageous since they do not require cryogenic temperatures and, therefore, bulky cooling systems. The overall detector area was increased by 4.5× by using CZT detectors in comparison to HPGe, while still maintaining an energy resolution of 2.26 keV at 59.5 keV. They determined that the spectral quality of their system was sufficient to separate liquids and crystalline substances while also providing enough spatially resolved scatter information to reconstruct 3D images of the bags’ contents [86].

Similarly, XRD methods were used by Rebuffel, Rinkel, et al. in [87] to detect explosive materials in an in-line baggage scanning configuration. A dual energy flat panel created by sandwiching a 0.5 mm thick $Gd_{2}O_{2}S$ layer with a 3.0 mm CsI layer was compared to an XRD system with CZT detectors arranged in a scatter configuration adjacent to the dual energy transmission detector. The transmission detector gathered radiographic images of the bag, while the scatter detectors collected spectroscopic information from the bag’s content. Both of these systems were evaluated separately to determine their effectiveness in determining explosive materials. Explosives behave similarly to plastics in terms of x-ray interaction thus the database generated by the authors was mainly comprised of high-density plastics such as Polyethylene (PE), Polyoxymethylene (POM), and Polytetrafluoroethylene (PTFE). A reference database was created by scanning known materials with known composition and electron density. The measured spectra were then compared to the spectra gathered from calibration materials. The diffraction method based on CZT detectors allowed for the discrimination of materials to a finer degree in comparison to transmission scintillator-based detectors. As an example they were able to discern water from hydrogen peroxide ($H_{2}O_{2}$) [87].

##### Illicit Substance Detection

An Energy Dispersive X-ray Diffraction (EDXRD) system was studied by the authors in [88]. The detector used was a single-pixel Amptek XR-100T CdTe detector with a crystal volume of 5 × 5 × $1\;{\mathrm{mm}}^{3}$. The energy resolution of the detector was 1.5 keV at a 122 keV (Co-57) peak. The x-ray tube was a Varian NDI-225-22 with a focal spot of 5.5 mm. A simulated traveler’s luggage experiment was conducted with a wooden box filled with a bag of flour, a block of Polymethyl methacrylate (PMMA), a pair of scissors, a bag of paracetamol, and a bag of $SiO_{2}$ [88]. This system proved that contraband detection is possible with an energy resolution of 5% FWHM at 29 keV within a 3-s detection time. Greenberg et al. proposed a multi-modal design to incorporate x-ray diffraction tomography and CT to detect illicit substances in luggage [89]. They tested their concept by combining a Smiths CTiX system inline with a coded aperture x-ray diffraction tomography (XRDT) system. The object would pass through the CT system and generate tomographs based on the CT density of the object. At this point any shape based features can be detected for illicit materials. Then, the object passes through the XRDT system where transmission radiographs and coded angle and energy dispersive scatter data is collected. The data is combined through a co-registration process, and then a correlation based classifier determines whether the object is a threat or not based on the updated information from the XRD data. Similarly, Greenberg et al. [89,90] proved that CT data gathered with current methods can be refined with energy information gathered from direct conversion detectors all within a 3-s detection time.

#### 3.1.7. Material Decomposition

The method of material decomposition has been explored to enhance material separation. The ability to individually count photons and bin them according to their energy allows for quantitative measurements to be inferred from tomographs. Although this method is not new to the medical field [91,92], the NDT&E field encompasses a wide range of materials in a single sample that benefit from this technique [93]. New areas in industrial X-ray imaging are using multi-energy techniques to enhance contrast and perform quantitative CT with the help of direct conversion detectors. The reduction of beam hardening artifacts through scatter reduction are enough to push for the adoption of direct conversion detectors in industrial X-ray CT [94].

Busi et al. [95] used a Multix ME100 sensor (CdTe) and performed material decomposition based on the collected spectra. The proposed method was based on tabulated attenuation data so there was no need for calibration between known materials. The driving factor in material characterization accuracy is the energy dependence of the attenuation coefficients and the number of energy bins used in the measurement. Since a direct conversion detector can measure the energy dependence of the linear attenuation coefficients directly, there is no need for calibration phantoms to be used in identifying unknown materials [95].

Buttacavoli et al. [96] utilized a CZT detector, developed by the Institute of Materials for Electronics and Magnetism (IMEM) in Parma, Italy, and Due2lab, for a food contamination detection system. This detector featured a 3.0 × 10.4 × 1.1 mm^2^ CZT crystal and a 32-pixel linear array with each pixel measuring 250 µm. The researchers employed a window-based energy selection method to identify the optimal energies that maximized the contrast-to-noise ratio (CNR) and differentiation between organic and inorganic materials. By applying this method, they enhanced the CNR of their images by 50% and successfully distinguished between steel, plastic, and coffee in their test samples.

#### 3.1.8. Density Measurements

Electron density and effective atomic number is needed to completely characterize a material. This is because materials may have varying electron densities while having effective atomic numbers that are near identical [97]. Jumanazarov et al. [97] determined electron density in reconstructed tomographs using a Multix ME100 detector with a 128-pixel linear array. Although their proposed method, the Spectral Algorithm for $\rho_{e}$ Estimation (SARE) [97], requires a set of calibration materials, on average they predicted electron density and effective atomic number within 1.2% and 2.4%, respectively.

#### 3.1.9. Archaeological NDT

Non-destructive techniques have been used countless times in determining the authenticity of paintings and artifacts [98,99,100]. Artifacts must be stored correctly and in order to determine the best storage method, the degradation state of the item must be investigated. In [101] the authors studied the degradation of historical beeswax seals to determine their best method of preservation. Gas chromatography can be used to determine the constituent chemicals of the individual seal, but it is a destructive process since the sample must be vaporized [102,103]. X-ray micro-tomography was chosen to analyze the samples non-destructively [101]. X-ray micro-radiography and micro-tomography were utilized to view the surface defects and internal defects of the sample. A large-area 300 µm thick silicon RTSD (14.3 × $7.15{\mathrm{mm}}^{2}$, 2560 × 1280 pixels) was created by combining 50 edgeless Timepix chips [73]. A magnification of 14 was achieved for the sample which resulted in an effective pixel size of 4 µm. This allowed the authors to measure micro-cavities and morphological structures as small as 5 µm without the destruction of the sample.

#### 3.1.10. Nuclear Non-Proliferation and Security

Nuclear waste drum inspection is highly important in nuclear waste characterization. The French Alternative Energies and Atomic Energy Commission (CEA) investigated the use of RTSD at higher energies (9 MeV) and compared the performance to a scintillator-based camera system [104,105]. The camera system consisted of a GADDOX screen coupled with a low-noise pixelated camera while the semiconductor detector consisted of 25 individual CdTe sensors, each measuring 0.8 mm × 15 mm, with a 25 mm crystal thickness [105]. The GADDOX scintillator was designed to be thin to decrease scintillation blur and automatically increase the spatial resolution. This caused a drop in detection efficiency at higher energies and an increase in noise. The GADDOX camera system produced an SNR of $10^{-3}$ while the multi-CdTe setup produced an SNR of 0.5. The high energy photon absorption efficiency of the CdTe crystal also allowed to increase the maximum scannable thickness of concrete from 60 cm to 160 cm [105]. Additional discussion on general gamma-ray imaging for application in arms control and nuclear non-proliferation can be found in [106,107,108,109,110].

#### 3.1.11. Quantifying the Amount of Uranium in Nuclear Fuel

Gilbert et al. [111,112] and Kasparek et al. [113] explored using CZT detectors to determine the mass of Uranium in Uranium Oxide powders using spectral radiography. The authors used a HEXITEC detector [114] which had a 20 × 20 × $1\;{\mathrm{mm}}^{3}$ CdTe crystal bump-bonded to an ASIC that contained 80 × 80 pixels. The detector offered an energy range from 4 to 200 keV. The standard deviation of their estimates were 0.62% and were compared to the Combined Procedure for Uranium Concentration and Enrichment Assay (COMPUCEA) method [115]. The COMPUCEA method provided a more accurate measurement with a standard deviation of 0.20%, but the method destroys the sample through powder dissolution [111]. An optimization model was used to estimate the areal density of Uranium and Oxygen separately. The estimated areal density was determined by deriving a system response function which accounted for detector efficiency loss due to charge-sharing removal and energy bin sensitivity. Total variation regularization was incorporated into their model to penalize sharp variations between neighboring pixels. This provided a more stable and accurate solution to the optimization problem leading to mass estimation errors below 1% [111].

### 3.2. Spectroscopy

#### 3.2.1. Explosive Detection

Much like the authors in [86,87], Crespy et al. [116] used the concept of XRD spectroscopy to test and expand the influence of many acquisition parameters in a scanning experiment, directly testing the performance and trade-offs associated with a HPGe detector against a CZT detector. The authors extended the work in [86,87] by establishing what they called a crystalline criterion. This criterion helped discern common materials that do not have a crystalline-like structure, such as toothpaste and coffee, to high explosives that do, such as trinitrotoluene (TNT). The crystalline criterion consisted of comparing areas under the curve of the collected energy spectra and a smooth fitted line. The area between the smooth curve and the raw spectra was calculated as the material crystalline quantity, while the amorphous quantity was the area below the smooth curve. The ratio of these two areas, defined as the crystalline criterion, was compared between explosives and common materials. The crystalline criterion allowed the authors to threshold against the detection of non-explosive materials, but a secondary criterion, defined as the similarity criterion, was needed to validate materials similar to high explosives. The similarity criterion was essentially the cross correlation between two independently captured spectra [116]. Briefly, the authors compared the two main peaks in the spectra, a large main peak and a smaller secondary peak, to lower false alarm rates. They determined that the two main peaks of explosive spectra occurred between $1\;{\mathrm{nm}}^{-1}$ and $1.6\;{\mathrm{nm}}^{-1}$ for HPGe and $1.2\;{\mathrm{nm}}^{-1}$ and $1.6\;{\mathrm{nm}}^{-1}$ for CZT. The three stage process to discern explosive materials was to (1) perform the crystalline criterion calculation, (2) compare the material to existing crystalline materials through the similarity criterion, and finally (3) perform spectral peak analysis to determine the chemical composition of the unknown material. This three-stage process is contingent on the accuracy of the detector. Two main peaks are resolvable using the CZT detector, but several peaks are possible to visualize only using a HPGe. In conclusion, HGPe detectors are more accurate, but CZT are more portable [116].

#### 3.2.2. Unmanned Aerial Vehicle Radiological Surveying

RTSDs have seen many uses in large-scale applications such as Unmanned Aerial Vehicle (UAV) radiation surveying. Vetter et al. [117,118,119] have investigated image fusion techniques paired with augmented reality to provide users with fast and precise radiation mapping, radiation hotspot localization, and decontamination verification.

Martin et al. [120] proposed the use of a Kromek GR1 CZT coplanar-grid radiation detector with $100\;{\mathrm{mm}}^{3}$ of volume. The small form factor allowed the authors to utilize UAV’s to perform higher resolution radiation maps (up to a meter scale) along with isotopic finger-printing with an energy resolution of 2% FWHM at 662 keV. Although a smaller detector volume is more efficient in carrying capacity, the smaller volume makes it more difficult to obtain adequate signal at the same altitude. This means that for the same signal at higher altitudes with a larger detector volume, the user will need to fly the UAV at a lower altitude. This could mean an alteration to the flight path would be necessary or the UAV is more susceptible to weather conditions [120]. The authors determined that the use of the CZT detector provided a low cost, rapid mobilizing, and completely autonomous solution for radiological surveys that can span areas up to $120,000\;m^{2}$ [120].

The authors in [121] created a visuo-haptic system that allowed users to manipulate a drone carrying a CZT detector using a haptic device. The system used a 5 × 6 × $20\;{\mathrm{mm}}^{3}$ drift strip detector with an dynamic energy range from 10 keV to 1.5 MeV. The user would receive a visual and haptic feedback during drone operation around a radiative material. The augmented reality representation of obstacles, the detected radioactive material, and the UAV appeared on the screen for the user. The authors noted that the main limitation of this system is that a single point camera was used which limited the available FOV of the environment [121]. The low power consumption and small form factor (0.3 kg) of the CZT detector allowed the authors to attach the detectors to a drone and transmit a full spectrum approximately every 2.2 s to the ground station [121]. For other developments of RTSD-based UAV systems in the last decade, we redirect the reader to [3,122] for further details.

#### 3.2.3. Harsh Environment Survey

##### Marine Environment

Remote sensing of radiation in harsh marine environments have been investigated using CZT detectors inside a Submarine Gamma Imager (SUGI) [123,124]. Radiation signatures underwater play a key role in national security, geological research, and nuclear accident response [123]. Aside from the harsh environment, water heavily scatters photons. The authors in [124] used an Integrated Detector Electronics AS (IDEAS) GDS-100 detector system with 4 GDS-10 modules. Each module consisted of a CZT crystal with 11 × 11 pixels per detector module and a crystal volume of 22 mm × 22 mm × 10 mm. Gas vents at the Alykes and the Paleochori beaches on the Milos islands off the coast of Greece were investigated. Spectra measurements at the seabed and various water depths were collected and analyzed. The authors field tested the enclosure and compared the spectral performance of the developed SUGI system to the fully characterized commercial µSPEC 4000 detector [124]. The average cps from baseline for the SIGU and µSPEC 4000 were 6 and 8, respectively, at various locations on both beaches. The authors concluded that the SUGI performed similarly to the commercial offering spectrally, and provided a gamma imaging capability to detect, localize, and identify radioactivity in an underwater environment [124].

##### Radiation Environment

Zhu et al. [125] used a CZT detector to characterize the leakage radiation given off an x-ray security inspection machine. The x-ray machine that was inspected was a CX100100D manufactured by NUCTECH. This inspection machine contained two independent x-ray sources with a lead curtain that provided shielding at the luggage opening. A GR1 CZT spectrometer developed by Kromek was used which contained a 10 mm × 10 mm × 10 mm CZT crystal. The authors investigated the influence of the lead curtain opening angle and the luggage size on the measured radiation dose 5 cm away from the opening of the inspection machine. The energy spectra gathered from the spectrometer was used to calculate the dose through the spectrum-dose conversion function G(E) [125,126]. The authors were able to produce dose rates with less than 2.5% deviation directly from the gathered spectra. This proved that CZT spectrometers can be utilized in predicting radiation dose in harsh radiation environments [125].

Parra et al. investigated the use of a Timepix3 detector to classify radioactive waste under tight time and spatial constraints in a nuclear decommissioning environment [73,127]. A 5 mm CdTe Timepix detector was characterized to determine the feasibility of using this sensor as a single-layer Compton camera. The electron mobility, resistivity, and energy resolution were assessed. The energy resolution was determined to be 16.3 keV for a Cs-137 photopeak where the optimal operating voltage was determined to be between −1300 to −2500 V. A localization resolution of 76 cm and 51 cm for Cs-137 and Co-60 was determined respectively [127].

Alam et al. [3] recently reviewed the advancements in CZT detector technology as effective ground-based, under-water and air-borne monitoring systems for nuclear power plant applications over the last decade.

#### 3.2.4. Food and Drug Inspection

##### Compacted Pill Inspection

Sub-pixel inspection techniques were used to determine the amount of metal contamination in powder pressed pills [39]. The detector used was a 2-mm thick CZT crystal that was bump bonded to a Timepix3 ASIC with a $55\;{\mathrm{µm}}^{2}$ pixel pitch [73]. The pixels were subdivided into a 3 × 3 grid creating a subpixel resolution of 18.3 × $18.3\;{\mathrm{µm}}^{2}$. Energy weighting was explored to integrate the images captured over the entire spectrum of energies from the x-ray source. Weighted schemes were explored to compare the methods used to highlight metal contaminants in an energy integrated image. Energy weights were determined using the photoelectric energy dependence, $E^{-3}$, the contrast to noise ratio (CNR) per energy bin, and the contrast to noise variance ratio (CNVR) per energy bin [39]. The CNVR energy weighting scheme provided a 2× increase in the integrated image CNR over the unweighted energy integrated image. This weighting method allowed for sub-pixel resolvability of heavy metal contaminants while not compromising on contrast in the resultant energy integrated image.

A Redlen CZT detector was used to validate the use of RTSDs in in-line food inspection within a conveyor belt system [128]. The detector used consisted in two detector modules for a total sensitive area of 7.9 × 190.1 mm with 24 × 576 pixels of 300 µm pitch. The authors claimed that the spectral imaging capabilities of the RTSD would allow for better detection of different types of materials from background. Several spectral images were captured by binning the energy range of interest (16–200 keV) into seven bins. Bin #2 which consisted of energies between 30 and 50 keV had the highest CNR among all energy ranges. This allowed for the separation between contaminants and background, such as stainless steel, glass, and Teflon [128].

##### Material Decomposition in Meat Inspection

Perion et al. [129] used a Multix ME100 detector module to separate meat, fat, and non-organic materials. The ME100 detector element consisted of 128 pixels arranged in a linear array with a CdTe crystal thickness of 2 mm. The pixel pitch was 0.8 mm with a sensitive area of 102.3 mm × 0.8 mm. The authors determined that fat quantification of 1% is possible through a three material decomposition method [130]. The authors also determined that 128 energy band separation aided in discriminating between materials especially at lower energies. The larger amount of energy bins increased the accuracy of the system by decreasing the false alarm rate by 3 × going from a two material decomposition to three [130], along with the mean relative error [129].

##### Heavy Metal Detection in Dried Fruit

Heavy metal detection through neutron activation analysis (NAA) [131] was investigated comparing CZT and HPGe detectors [132,133]. Foley et al. [132] investigated arsenic salt concentrations diluted in water samples to determine the capability of a 5 mm × 5 mm × 5 mm CZT detector developed by Kromek. The crystal contained platinum capture contacts and was connected to an eV-550 preamplifier unit. A Canberra DSA-1000 multichannel analyzer was used with the GENIE2000 spectroscopy software. The minimum detectable activity (MDA) was the factor of comparison between the CZT and HPGe detectors. The MDA for the CZT detector was $1.8\;\times \;10^{-5}$ mCi while the MDA for the HPGe detector was $1.8\;\times \;10^{-6}$. The magnitude difference was concluded to be from the smaller detector volume of the CZT detector. The volume of the HPGe crystal was over 10 × which contributed to the increased confidence in detecting smaller traces of heavy metals in the samples. The authors concluded that CZT has the potential to be used as a portable NAA detector since room temperature operations is possible [132].

#### 3.2.5. Density Measurements

Absolute density measurements have been performed for in-line wood inspection using several single-pixel CZT detectors and Hyperspectral X-ray Absorptiometry [134,135]. The detector contained a CZT crystal by Redlen and had a volume of 4.1 × 4.1 × $2.5\;{\mathrm{mm}}^{3}$. The authors used Beer’s law of attenuation to perform a best fit of the transformed spectral data. They determined the density of wood and graphite within 2% of the expected density values.

#### 3.2.6. Nuclear Non-Proliferation and Security

##### Waste Barrel Identification

Magnox nuclear reactor waste was simulated in an experiment to observe the use of a robotic arm (Motoman MA1400 dual-arm robotic manipulator (Yaskawa Motoman, Miamisburg, OH, USA) with a CZT detector to generate a count rate map [136]. A Kromek GR1-A+ with a CZT detector size of $1\;{\mathrm{cm}}^{3}$ was used. The movement path and speed of the robotic arm was investigated to determine the optimal scanning strategy that provided adequate counts to generate count maps and determine radiation hot spots. Co-60 and Cs-137 point sources were used to simulate radioactive waste to determine the MDA of the system. The MDA ranged from 5–10 kBq at a distance of 0.1 cm from the source with layers of water, concrete, and steel [136]. The authors also determined that a scanning speed of 5 cm/s in a 50 cm × 50 cm square area provided adequate counts to generate radiation maps. The portability and detector count speed allowed the authors to create a mobile setup to identify simulated waste barrels.

The Atomic Energy and Alternative Energies Commission Institute of research into the fundamental laws of the Universe (CEA IRFU) investigated the development and use of a gamma camera consisting of a coded aperture and a Compton imaging system for use in nuclear industry applications [137]. The new system, named Spid-X, aimed to create real-time isotope identification through the improved readout speed of a RTSD and a trained Neural Network. The Spid-X detector consisted of a 16 × 16 pixel ASIC with 800 µm pixel pitch and a 2 mm thick CdTe crystal. The authors verified each system component from the automatic identification of radioisotopes, measurements in the presence of multiple detectors, imaging, and dosimetry at the camera level [137]. The automatic isotope identification showed to identify an unobstructed Am-241 source with an activity of 407 kBq at 1 m away. A timed study was then performed to compare the known methods with Compton imaging. The direct back projection, various iterative methods such as iterative Bayesian approaches, and deep learning methods were investigated and compared. The deep learning method was trained with various Monte Carlo simulations with varying isotope locations and their respective back projected results to develop a learning database [138]. The authors calculated the interaction position in 30 ms providing the basis to achieve near real-time radioactive source detection [137].

##### Nuclear Fuel Enrichment Detection

An indirect method to measure Plutonium enrichment levels in spent nuclear fuel was proposed by Seo et al. in [139]. The detector used was an eV-CPG gamma detector manufactured by Kromek which consisted of a coplanar grid CZT detector with a detectable volume of 10 × 10 × $10\;{\mathrm{mm}}^{3}$. The detector was placed underwater near the nuclear fuel rod under operation. The authors deduced that the indirect measurement of the amount of fuel left in the fuel rod was related to the activity ratio between Cs-134 and Cs-137. The activity ratio was obtained through gamma spectroscopy and plotted against predicted burn up calculated from the ORIGEN computer code [140]. The authors separately validated the ORIGEN burn up measurements with known Destructive Assay methods and corrected for the difference in measured and calculated activity levels of Cs-134 and Cs-137 [139]. The authors showed a linear fit between the recorded activity ratio and burn up which could be used in near-real time accountancy at pyroprocessing facilities or during transport/storage at interim storage facilities. This was made possible due to the CZT detectors reasonable energy resolution and better portability than HPGe detectors [139].

Morteau and Berndt [141] used a CZT detector developed by RITEC to determine the Uranium enrichment in nuclear fuel. The hemispherical CZT detector contained a crystal with a volume of 15 × 15 × $7.5\;{\mathrm{mm}}^{3}$ and was used to determine Uranium enrichment ranging from 0.31% to 94.1%. Although the detector experienced spectral peak degradation because of electrical circuitry noise, the use of a CZT detector is feasible in determining the Uranium enrichment through shielding ranging from 2 mm of Aluminum to 16 mm of steel within a counting time of 100 s [141].

##### Nuclear Aerosol Detection

Ranjbar et al. [142] developed a CZT detector to detect Xenon isotopes (Xe-131m, Xe-133m, Xe-133, and Xe-135) in the form of aerosols. These isotopes are a large determining factor in verifying if an explosion is nuclear in nature [142,143,144]. The authors compared their design to the scintillation based detection systems in place by the Comprehensive Nuclear-Test-Ban Treaty Organization (CTBTO) [145,146,147]. The plastic scintillators used in current systems were cited to have problems in detecting both gamma rays and beta particles due to the sandwiched scintillator setup. The plastic scintillators experienced a memory effect that degrades the minimum detectable counts (MDC) [148]. The CZT crystals used were 10 × 10 × $10\;{\mathrm{mm}}^{3}$ and were provided by Redlen Technologies. The MDC of the CZT detector was 1.47 ± $0.05\;{\mathrm{mBq/m}}^{3}$. The authors reported that at the 250 keV (Xe-135) gamma peak, they obtained an improvement ranging from 54% to 77% compared to current scintillator detectors [142]. The authors detailed that a larger gas test chamber and increasing the number of CZT detectors would increase the geometric detection efficiency decreasing the MDC to less than $1\;{\mathrm{mBq/m}}^{3}$.

Similarly, a dual detector device was designed to detect radiation above and below the instrument to measure gamma radiation incidents [33]. The first CZT detector would be facing upward to gather counts from the air and the second would be directed towards the ground to detect radiation in the soil. The CZT detector modules were 15 mm × 15 mm with a 7.5 mm thickness and an energy resolution of 3.45% [33]. The upper CZT detector unit had a 6 mm thick polyethylene hemisphere that acted as the neutron moderator. The upper detector would detect fast neutrons as well as gamma rays, while the bottom detector would just record gamma rays. The subtraction of the two spectra would provide the incident neutron spectrum. Cf-252 (neutron emitter) and Cs-137 (gamma emitter) were used to test the capabilities of the detector. The neutron capture performance of Cd-113 and the energy resolution performance of the CZT crystal allowed the development of a portable neutron and gamma detecting device [33].

Kurvinen et al. [149] designed an all-in-one system that would be mounted to a UAV to detect radioisotopes in radioactive plumes. The goal was to detect the radioactive dose, localize the the radioactive plume, identify and gather gamma emitting radionuclides. Multiple detectors were used in one device to accomplish these goals. A custom CZT detector was used to identify gamma-emitters because of its superior energy resolution while the NaI scintillator detector was used to localize the radioactive plume, and a Geiger-Mueller counter (GM-counter) was used to determine the dose rate. The size of the CZT crystal was 5 × 5 × $5\;{\mathrm{mm}}^{3}$ compared to a 150 mm × 100 mm cylindrical crystal. The GM-counter was an all enclosed system that had a detectable range from 0.01 µSv/h to 10 Sv/h [149]. Paper glass-fiber and carbon air filters captured air samples as the UAV traveled through a radioactive plume. Then, the radiation detectors absorbed the radiation given off the accumulants in the filters. The authors observed $100\;{\mathrm{Bq/m}}^{3}$ of I-131 in 20 min of flight time in the active plume [149]. Other low energy gamma emitters, such as Ru-103 and Ce-141, accumulated on the filters and were estimated to have an activity between 20–$400\;{\mathrm{Bq/m}}^{3}$ [149]. This system utilized multiple detectors for their strengths and provided enough redundancy to make a more robust detection system in a radiative harsh environment.

##### Special Nuclear Material Detection

The detection of shielded Special Nuclear Material (SNM) is difficult and of great importance to national security. Gadey et al. [150] extended the design of a radioxenon detection system to include stilbene gas cell coupled with a coplanar CZT detector similar to the design proposed in [142]. The decay scheme of radioxenon isotopes are of primary interest to Comprehensive-Nuclear-Test ban treaty (CTBT) because they are fission products of Uranium [143,144]. The decay scheme of these radioisotopes is a gamma-beta particle coincident reaction. Previous detector designs accomplished this by coupling a plastic scintillator to detect beta particles while a NaI scintillator would detect gamma-rays [151]. Gadey et al. [150] used a gas test cell made from stilbene coupled with a silicon photomultiplier tube (SiPM) to detect beta particles, and a coplanar CZT detector with a detector volume of 10 × 10 × $10\;{\mathrm{mm}}^{3}$ designed by Redlen Technologies to detect gamma rays [150]. The detector produced two spectra and were combined to form a spectral surface, known as a coincidence plot. The coincident plot would produce the back scatter coincidence line which would be constant in energy and identify specific radioxenon isotopes based on tabulated data. A test measurement was performed with a Cs-137 source and was calibrated to its 662 keV peak. The MDC of the stillbene-CZT detector was 0.483 ± $0.006\;{\mathrm{mBq/m}}^{3}$ compared the two element CZT (TECZT) which was 1.45 ± $0.06\;{\mathrm{mBq/m}}^{3}$ when detecting Xe-131m [142,150].

An indirect approach in determining SNM was investigated to correlate the atomic number and mass thickness of intervening materials to Uranium and Plutonium spectral measurements [152]. A radiation detector containing four 2 × 2 × $1.5\;{\mathrm{cm}}^{3}$ three-dimensional position-sensitive CZT detectors was jointly developed by IDEAS and the University of Michigan. The proposed algorithm estimated the effective atomic number using various gamma-ray lines in conjunction with Compton scatter to photoelectric ratios in shielding materials. The standard deviation of the estimated areal density was $1.3\;{\mathrm{g/cm}}^{2}$ and the estimated atomic number of the shield was within range of the true value for steel equivalent intervening material [152]. The authors noted that estimate accuracy would be improved as the photo peak counts improve. Improvements in energy resolution would also reduce the estimate uncertainty due to the increased energy separation between photo peak events and forward scattering events.

Dual particle imaging was investigated for the characterization and detection of SNM [153]. The detector was composed of a 3 × 3 array of pixelated CZT detectors each with a volume of 2 × 2 × $1.5\;{\mathrm{mm}}^{3}$. Each detector was reported to have an energy resolution of 0.35% FWHM at 662 keV [153]. The neutron gamma dual particle imaging demonstrated that Plutonium Oxide can be located through two detector events. They demonstrated that through neutron imaging the correct source location was determined as an alternative signature to gamma-ray emission, in case the gamma-rays are obscured by shielding or other intervening material. The authors also induced fission in Uranium with varying enrichment levels and observed that the delayed neutron emission time profiles can provide isotopic composition of Uranium contained materials [153].

## 4. Discussion and Conclusions

This review shows that RTSDs and specifically Cd(Zn)Te detectors have gained attention in various NDT&E applications. From national security and threat detection to mechanical testing, these detectors showcase their ability to provide high-resolution images and valuable spectroscopic information. Key advantages highlighted include: (1) *High-resolution Imaging:* Cd(Zn)Te detectors enable high-resolution imaging both in terms of spatial and temporal resolution, allowing for the detection of subtle defects and the visualization of dynamic processes. This is exemplified by applications such as mechanical defect propagation and archaeological NDT [72,102]. (2)*Superior Spectroscopic Capabilities:* The ability to perform energy-resolved measurements provides valuable insights into material composition and allows for material discrimination. This translates in an improved accuracy and sensitivity, that enables quantitative measurements. For example, separating uranium and oxygen percent compositions of uranium oxide pellets in nuclear fuel and nuclear waste characterization [113,136,137]. (3) *Versatility and Portability:* Cd(Zn)Te detectors operate at room temperature, eliminating the need for bulky and expensive cooling systems. They have been successfully integrated into a wide range of systems due to their compact size and low power consumption, especially in the case of portable devices for field inspections, allowing for on-site and real-time data analysis of automotive inline welding inspection and harsh radiation environmental surveying, for inspections of constrained and confined environments such as pipe weld inspection, or for the study of material identification using Compton imaging techniques at a beam line facility [29,123,154]. This adaptability underscores their potential to address diverse NDT&E challenges. On the other hand, the development of large-scale systems still lags behind due to the complex manufacturability of large single Cd(Zn)Te crystals [46].

While in medical imaging the direct competitors are scintillator detectors [155], in NDT&E HPGe represents the gold standard, but many applications recognize the better portability of Cd(Zn)Te [116,139]. In applications where compactness is not a driving factor, another trend consists in combining different detectors (scintillator, HPGe, Cd(Zn)Te) in a hybrid setup, capitalizing on their individual strengths and providing sufficient redundancy to create a more robust and accurate detection system [149,150].

Future directions for Cd(Zn)Te detectors in NDT&E include further improvements in crystal growth techniques to produce larger, cheaper and higher-quality crystals that will facilitate their widespread adoption. Similarly to the medical industry, this will enable the development of larger-area detectors with increased detection efficiency. Larger FOV stationary detectors would allow for larger objects to be imaged, increase the maximum magnification of objects allowing to observe finer details in radiographs/tomographs, and increase the detector’s geometric efficiency. On the other side, it will also facilitate the development and use of portable and cost-effective CZT-based NDT&E systems making this technology more accessible to a wider range of industries.

Advancements in signal processing and data analysis techniques will enhance the capabilities of Cd(Zn)Te-based imaging systems, allowing for more accurate and quantitative inspections. The ability to simultaneously gather spectral and imaging information while accounting for signal loss such as charge sharing will provide the foundation to perform quantitative measurements. The promise of wider adoption of RTSDs can be seen in the development of IEEE standards that regulate the performance, development, and implementation of these types of detection systems [156].

It is worth noting that Cd(Zn)Te detectors could facilitate the development of autonomous solutions for detecting radiation in harsh or hard-to-reach environments, both in nuclear and non-nuclear settings. For example, 3D position-sensitive CZT detectors assure more accurate 3D localization of radiation events and radioisotope identification, leading to improved data quality and reduced measurement uncertainties that could be used for detecting unwanted nuclear material holdup in nuclear reactor coolant pipes, SNM in cargo containers, and detecting dirty bombs [108,109,157].

In this paper we have discussed the key performance parameters that characterize an RTSD system, degradation factors that harm detector performance, and discussed state-of-the-art applications of RTSDs to exemplify their spectroscopic and imaging performance. While challenges such as cost and slow fabrication remain, the continued development and refinement of Cd(Zn)Te detector technology including the need for further advancements in data analysis and processing holds significant promise for advancing NDT&E.

## Figures and Tables

**Table 1:** Summary of state-of-the-art CZT detector energy resolution values with associated Fano noise [15,16,17].

Spectral Energy (keV)	Energy Resolution FWHM (keV)	Fano Noise (keV)
59	0.576	0.444
122	1.170	0.617
662	3.970	2.200

## Data Availability

No new data were created or analyzed in this study. Data sharing is not applicable to this article.

## Abbreviations

The following abbreviations are used in this manuscript:

ASIC	Application-Specific Integrated Circuit
CEA	The French Alternative Energies and Atomic Energy Commission
IRFU	Institute of Research Into The Fundamental Laws of the Universe
CNVR	Contrast to Noise Variance Ratio
COMPUCEA	Combined Procedure for Uranium Concentration and Enrichment Assay
cps	Counts Per Second
CSA	Charge-Sharing Addition
CSD	Charge-Sharing Discrimination
CT	Computed Tomography
CTBT	Comprehensive Nuclear-Test-Ban Treaty
CTBTO	Comprehensive Nuclear-Test-Ban Treaty Organization
CUI	Corrosion Under Insulation
CZT	Cadmium Zinc Telluride
DOI	Depth of Interaction
EDXRD	Energy Dispersive X-ray Diffraction
FOV	Field of View
fps	Frames Per Second
FWHM	Full Width at Half Maximum
GADDOX	Gadolinium Oxysulfide
HPGe	High Purity Germanium
IQI	Image Quality Indicators
MDA	Minimum Detectable Activity
MDC	Minimum Detectable Counts
NAA	Neutron Activation Analysis
NDT&E	Non-Destructive Testing and Evaluation
PE	Polyethylene
PMMA	Polymethyl methacrylate
PMT	Photomultiplier Tube
POM	Polyoxymethylene
PTFE	Polytetrafluoroethylene
RTSD	Room Temperature Semiconductor Detector
SNM	Special Nuclear Material
SPECT	Single Photon Emission Computed Tomography
SUGI	Submarine Gamma Imager
TECZT	Two Element CZT
TNT	Trinitrotoluene
XRD	X-ray Diffraction
